# Availability of emergency neonatal care in eight districts of Karnataka state, southern India: a cross-sectional study

**DOI:** 10.1186/s12913-015-1126-3

**Published:** 2015-10-06

**Authors:** Prem K. Mony, Krishnamurthy Jayanna, Swarnarekha Bhat, Suman V Rao, Maryann Crockett, Lisa Avery, BM Ramesh, Stephen Moses, James Blanchard

**Affiliations:** St John’s National Academy of Health Sciences, Bangalore, India; Karnataka Health Promotion Trust, Bangalore, India; Department of Community Health Sciences, University of Manitoba, Winnipeg, Canada; Division of Epidemiology & Population Health, St John’s Medical College &Research Institute, St John’s National Academy of Health Sciences, 100 feet road, Koramangala, Bangalore, 560034 India

**Keywords:** Emergency neonatal care, Health services, India

## Abstract

**Background:**

Emergency Neonatal Care (EmNC) is an important service for the health and survival of newborns. The objective of our study was to assess the availability of emergency neonatal care services in the north-eastern region of Karnataka state in India.

**Methods:**

We undertook a cross-sectional epidemiologic study in the year 2010. We assessed the provision of eight life-saving ‘signal functions’ (Comprehensive EmNC) or at least five ‘signal functions’ (Basic EmNC) by self-reporting through a structured questionnaire, coupled with verification by direct observation for presence of drugs and equipment in the prior three months. The assessment was undertaken in 443 government and 422 private healthcare facilities of eight districts of Karnataka.

**Results:**

There was an average of 3.6 EmNC facilities available per 500,000 population for the entire region. Only three out of eight districts and 10 of 42 sub-districts in the region had the recommended [greater than or equal to 5] EmNC facilities per 500,000. Further, over 95 % of CEmNC facilities and 88 % of BEmNC facilities were within the private sector. About 80 % of government hospitals at district and sub-district levels did not have EmNC capability.

**Conclusions:**

This study demonstrates the feasibility of using a simple assessment tool to measure health facility availability of life-saving services for newborn care. EmNC availability was seen to be suboptimal at the regional, district and sub-district levels within the northern part of Karnataka state. There is a need to improve availability of emergency newborn care in health facilities, with special emphasis on equity at population level.

## Background

While maternal and postneonatal mortality have been declining globally at over 3 % per year since 1990, reduction in neonatal mortality has been lagging behind at about 1.8 % per year during this period [1]. This burden of disease due to neonatal conditions is also concentrated disproportionately in few settings. A cluster of clinically avoidable conditions (complications of low birth weight/prematurity, intra-partum problems including birth asphyxia, and neonatal infections) contribute to the bulk of the neonatal mortality in India [2]. While both distal (socioeconomic) and proximate (health system and individual) determinants play a role in improving child survival [3], the latter need to be urgently addressed to reduce neonatal mortality that has not been falling fast enough in India. Emergency obstetric and neonatal care (EmONC), comprising a set of life-saving clinical functions, has been proposed by several organizations to improve maternal and neonatal health outcomes. While the availability of emergency obstetric care (EmOC) has been studied in some detail in south Asia [4–7], emergency neonatal care (EmNC) has been studied less often, owing in part to a lack of consensus on the signal functions to be assessed. While earlier a single signal function (neonatal resuscitation) had been considered [8], more recently nine signal functions have been proposed [9]. Hence there is a need to study the provision of these functions at both the hospital-level as well as the population-level. The objective of our study was to assess the availability of emergency neonatal care services in the northeastern region of Karnataka state in India.

## Methods

### Study design and setting

**Fig. 1:**
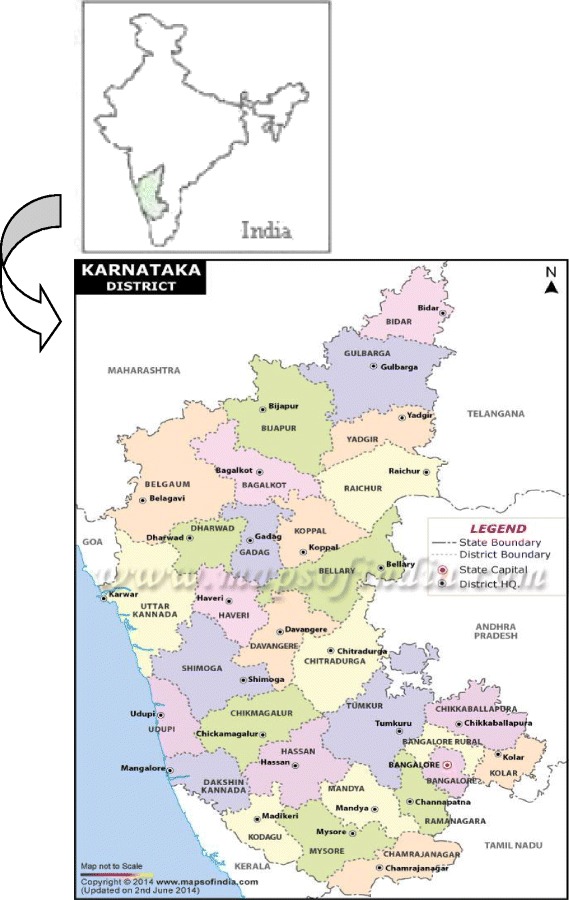
Map of the northeastern study districts within Karnataka state, India

The estimates of neonatal and early neonatal mortality rates for the state of Karnataka in the year 2010 were 25 and 22 per 1000 live-births respectively [10]. The health indicators for Karnataka are lower than the national average [eg. neonatal mortality rate (NMR) = 29/1000] and lie mid-way between the states of Kerala (NMR = 8/1000) and Tamil Nadu (15/1000) at one end and the north Indian states (NMR of Madhya Pradesh & Odisha = 40/1000) at the other [11]. This cross-sectional epidemiological study was conducted in eight northeastern districts (Bagalkot, Bellary, Koppal, Raichur, Yadgir, Gulbarga, Bijapur & Bidar) of Karnataka state in southern India (Fig. 1). The details are outlined elsewhere [7]. Briefly, this region, with 42 sub-districts (*taluks*), has a quarter of the state's total population of 60 million [7]. Compared to the rest of the state, the health indicators of this region are suboptimal with a relatively weaker health system and chronic staffing shortages [12]. As part of a 5-year technical support project (2010–2015) assisting the Government of Karnataka in strengthening maternal, neonatal and child health (MNCH) care in this region, we carried out a facility assessment and service availability survey (in antepartum, intrapartum, postpartum, newborn and child health domains) of all health facilities in this region during the year 2010 [7].

### Study population

Of a total of 3005 health facilities, 444 government facilities and 490 private facilities were potentially eligible study facilities since they were‘24/7’facilities (open 24-hours-a-day and 7-days-a-week). For 40 private hospitals, the bed-number was recorded as <30 but the exact number could not be ascertained. One government primary health centre (PHC) and 68 private hospitals refused to participate in the study. The remaining health facilities were divided into two groups. District hospitals (*n* = 8) and *taluk* hospitals (*n* = 34) from the government sector, and hospitals with >30 beds (*n* = 42) in the private sector were categorized as ‘large’ hospitals; and community health centres (CHCs, *n* = 69) and primary health centres (*n* = 332) from the government sector, and hospitals with ≤30 beds (*n* = 380) from the private sector were categorized as ‘small’ hospitals. Thus a total of 443 government and 422 private facilities were analyzed in this study. Less than two percent of private hospitals were not-for-profit hospitals and hence no sub-group analysis was undertaken.

### Study instrument

We developed a tool to cover the signal functions critical for neonatal survival (Table 1). Subsequently we grouped them into three categories based on a local adaptation of guidelines recommended by Gabrysch and others [9]. Corticosteroid use in preterm labour; kangaroo mother care (KMC) for small/preterm babies; bag-and-mask resuscitation; injectable antibiotics for mothers and newborns; and alternative feeding (tube/spoon feeding) were considered the five Basic Emergency Neonatal Care (BEmNC) services. Neonatal intubation; use of intravenous fluids; and oxygen administration were considered the three additional Comprehensive Emergency Neonatal Care (CEmNC) services. Thermal protection, early and exclusive breastfeeding, and hygienic cord care were considered routine essential newborn care. Health facilities were classified as CEmNC facilities (if providing all eight signal functions) or BEmNC facilities (if providing only the five basic signal functions) based on self-reported, continuous performance of signal functions in the previous 3-month period coupled with verification by record reviews and direct observation for availability of drugs, equipment and consumables required to perform the signal function. Record reviews of admission registers maintained in the newborn care units of these facilities were undertaken to verify hospitalization of newborns with complications such as low birth weight, birth asphyxia or neonatal sepsis in the previous three months. Suboptimal completeness and quality of documentation of individual case-sheets however precluded their use for any cross-verification. Stock registers of drugs and consumables were reviewed for documentation of any ‘stock-out’ in the previous quarter. A few critical equipment such as (i) bag and masks (for normal birth weight and low birth weight/preterm babies) for neonatal resuscitation; (ii) corticosteroids; (iii) oxygen cylinder with gauge; (iv) gastric feeding tube; and (v) intravenous fluids and infusion set, were considered mandatory at the time of the direct observation visits to corroborate a given signal function. So for instance, for bag-and-mask resuscitation to be included as a service offered by a given health facility, the bag and masks had to be available throughout the prior 3-month period as well as on the day of the research team’s visit. For antibiotics however, if the service was reported as being available by the healthcare provider but the drug register showed stock-outs or if it was unavailable on the day of the research team’s visit, it was accepted as available since it was a common practice for it to be procured on a *pro re nata* basis from private pharmacies in the same town as needed.

**Table 1:** Definitions of Essential and Emergency (Basic & Comprehensive) Neonatal Care services (adapted from [9]) and their recommended geographic distribution [8]

Routine Essential Newborn Care	Emergency Neonatal Care (EmNC) Services	
	Basic Emergency Neonatal Care (BEmNC)	Comprehensive Emergency Neonatal Care (CEmNC)
1. Thermal protection	1. Corticosteroids (in preterm labour)	✓ all BEmNC
2. Early & exclusive breastfeeding	2. Kangaroo Mother Care (KMC) for small/preterm babies	+
3. Hygienic cord care	3. Bag-and-mask resuscitation	6. Neonatal intubation
	4. Injectable antibiotics (for mother and newborn)	7. Intravenous fluids
	5. Alternative feeding (tube/spoon feeding)	8. Oxygen administration

Recommended geographic distribution (minimum): One CEmNC facility & four BEmNC facilities per 500,000 people

### Data collection, entry and analysis

Data collection was carried out during May to August 2010. Thirty five research assistants were recruited and trained over a period of 10 days during May 2010 to be the study interviewers. Fifteen of them with master’s level qualification visited the larger hospitals and the remaining twenty with bachelor’s level qualification visited the smaller hospitals. They collected information through a combination of face-to-face interviews with the facility staff (doctor/nurse/pharmacist), review of hospital records and direct observation. All questionnaire data, record reviews and direct observation of facilities were entered and analyzed using SPSS software (Version 18.0, Chicago, USA).

For analysis, EmNC by facility and by population were calculated. The population of the study districts for the year 2010 was calculated as the inter-censal estimates from the 2001 and 2011 census years’ data [13, 14]. The densities of BEmNC and CEmNC facilities were calculated per 500,000 population along the lines of definitions available for emergency obstetric and neonatal care distribution [8] – that is, a recommended minimum of one CEmNC and four BEmNC facilities per 500,000 persons (Table 1). For analysis, ‘any BEmNC’ means including facilities offering either BEmNC or CEmNC while ‘BEmNC-alone’ means BEmNC availability without CEmNC availability.

### Ethical and regulatory considerations

Regulatory permission for the study was obtained from the state wing officials of the National Health Mission for Karnataka and the Department of Health of the Government of Karnataka. Ethical clearance for the study was obtained from the Institutional Ethics Review Boards (IERBs) at St John’s Medical College, Bangalore, India and the University of Manitoba, Winnipeg, Canada. Written informed consent was provided by the chief medical officer of each participating facility.

## Results

Overall, there were a total of 5.7 hospitals per 100,000 population (public = 3.0, private = 2.7) in the study region. The distribution of the 443 public and 422 private health facilities (functioning “24/7”) per 100,000 population in the eight districts was as follows: Bellary (public = 2.7, private = 2.5); Bagalkot (2.8, 5.5); Raichur (3.0, 2.1); Bijapur (2.3, 3.5); Bidar (2.8, 1.8); Gulbarga (3.1, 2.7); Koppal (3.7, 1.8); and Yadgir (3.5, 1.5).

Out of the total of 865 health facilities surveyed, 95 (11 %) were CEmNC facilities and an additional 29 (3.3 %) were BEmNC-only facilities. The proportion of government and private hospitals offering comprehensive or basic emergency newborn care services is shown in Table 2. Availability of emergency newborn care was seen to improve by size of hospital in both the public and private sectors (Table 2). A substantially greater proportion (22 %) of private hospitals offered CEmNC services compared to government hospitals (0.5 %); similarly, proportion of private hospitals that offered BEmNC services (26 %) were substantially higher than that in the government sector (3 %); these differences were statistically significant. The remainder of the government and private facilities offered routine newborn care predominantly.

**Table 2:** Proportion of government and private facilities offering comprehensive or basic emergency newborn care services, northeastern Karnataka, 2010

Sector	Government		Private		Significance
Service	Category^a^	*n* (%)	Category	*n* (%)	Z-score; p-value
CEmNC	PHC (N = 332)	0	1-6 beds (N = 114)	8 (7 %)	
	--		1-30 beds^b^ (N = 40)	5 (13 %)	
	CHC (N =69)	0	7-30 beds (N = 226)	57 (25 %)	
	TH (N = 34)	0	31-50 beds (N = 31)	15 (48 %)	
	DH (N = 8)	2 (25 %)	>50 beds (N = 11)	8 (73 %)	
	*Subtotal* (N =443)	2 (0.5 %)	*Subtotal* (N =422)	93(22 %)	103.2; <0.001
BEmNC	PHC (N = 332)	0	1-6 beds (N = 114)	11 (10 %)	
	--		1-30 beds^b^ (N = 40)	6 (15 %)	
	CHC (N =69)	6 (9 %)	7-30 beds (N = 226)	69 (31 %)	
	TH (N = 34)	4 (12 %)	31-50 beds (N = 31)	16 (52 %)	
	DH (N = 8)	5 (63 %)	>50 beds (N = 11)	8 (73 %)	
	*Subtotal* (N =443)	15 (3 %)	*Subtotal* (N =422)	110 (26 %)	90.1; <0.001

^a^ DH = District hospital; TH = *Taluk* hospital; CHC = Community health centre; PHC = primary health centre; CEmNC = Comprehensive Emergency Neonatal Care; BEmNC = Basic Emergency Neonatal Care [all CEmNC facilities are also BEmNC facilities in this table];

^b^For 40 private hospitals, the total number of beds was listed as <30 but the exact number could not be ascertained

**Fig. 2:**
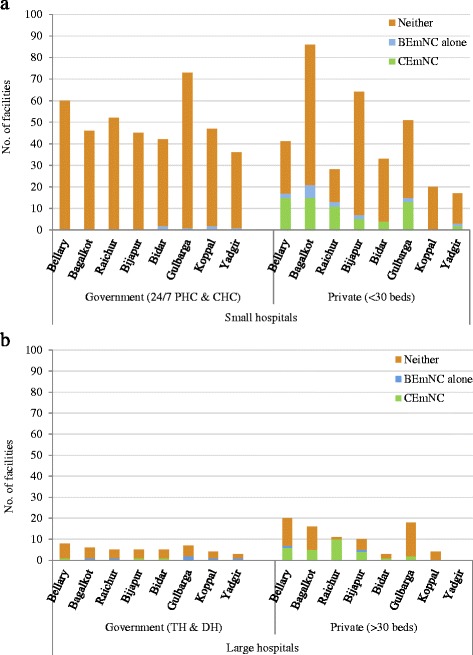
**a**. Proportion of ‘small’ health facilities providing basic or comprehensive emergency neonatal care services by sector and district, northeastern Karnataka (2010). **b**. Proportion of ‘large’ health facilities providing basic or comprehensive emergency newborn care services by sector and district, northeastern Karnataka (2010)

When examined by district, we observed that among the small hospitals (with bed-strength <30), none of the government hospitals offered CEmNC in the eight districts, while in the private sector CEmNC availability ranged from 0 % to 31 % (5/16; Bagalkot district) of facilities. Any BEmNC availability ranged from 0 % to 5 % (2/42; Bidar district) in the government sector and from 0 % to 46 % (13/28; Raichur district) in the private sector (Fig. 2a). Among the large hospitals (with bed-strength >30), CEmNC availability ranged from 0 % to 20 % (1/5; Bidar and Bijapur districts) in the government sector, and from 0 % to 91 % (10/11; Raichur district) in the private sector while any BEmNC availability ranged from 14 % (1/7; Bellary district) to 33 % (Yadgir district) in the government sector and from 0 % to 91 % (Raichur district) in the private sector (Fig. 2b).

**Fig. 3:**
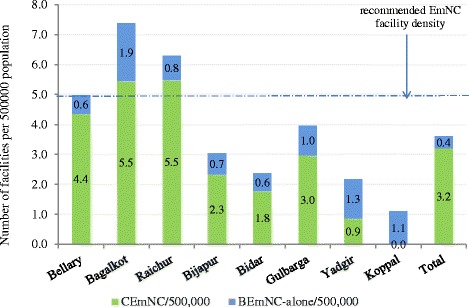
Distribution of emergency newborn care service availability by district, northeastern Karnataka (2010)

Overall, the study area had 3.6 EmNC facilities per 500,000 population, with only three of the eight districts having ≥5 EmNC facilities per 500,000 population (Fig. 3). This was largely driven by the contribution of the private sector, with nearly 88 % of all BEmNC facilities alone (or 60 % of BEmNC-alone facilities) and 97 % of all CEmNC facilities being in the private sector. At the *taluk* level, only ten out of 42 *taluks* had ≥5 EmNC facilities per 500,000 population (data not shown).

Across the region, for every facility offering only BEmNC services, there were eight facilities offering CEmNC services. It was also noted that as the availability of newborn care services improved across districts, the ratio of BEmNC:CEmNC facilities reversed (from 1.1:0 in Koppal district to 1:7 in Bellary district). In other words, districts with higher numbers of EmNC facilities overall also had higher proportions that were CEmNC.

Key deficiencies were noted in the large government hospitals that prevented them from being EmNC centres. Of the district hospitals, a little over 40 % were not offering corticosteroids for preterm labour, were not providing intravenous fluids to neonates and were not providing intubation for neonates. Of the *taluk* hospitals, 70 % were not providing alternate feeding or intravenous fluids, 50 % were not providing corticosteroids and only a third were providing bag-and-mask ventilation in room air, oxygen, and parenteral antibiotics for neonatal sepsis.

## Discussion

As India and other countries make their final push towards achieving their Millennium Development Goals (MDGs) and look to work in the post-2015 era, progress in reducing neonatal mortality is an important frontier. This study aids in the understanding of the availability of emergency care services for common neonatal complications at a sub-national level in India. The tool and methodology that we used was based on a local adaptation of that recommended earlier [9]. The overall availability of EmNC services in this region of India was suboptimal with less than 5 EmNC facilities per 500,000 population. Furthermore, the availability of these services was severely constrained in two domains: geographic and economic. Only three out of eight districts (and 10 out of 42 sub-districts) in this region had adequate numbers of EmNC facilities. Further, since over 95 % of CemNC facilities and 88 % of all BemNC facilities belonged to the fee-for-service private sector, and a large proportion of the local population in the region can ill-afford out-of-pocket expenditure [15], the majority of these services remain inaccessible to most families with newborns requiring these life-saving interventions.

Even though there was a wide network of multi-tiered government sector health facilities in the region, a vast majority (>97 %) of them functioned sub-optimally, by being unable to offer basic or comprehensive emergency newborn care services. This could potentially be due to resource shortfalls in human resources and/or capital/material. Under human resources, in our study area it was seen that it was due to both shortage of staff as well as serious gaps in their knowledge and skills. For example, we found that only 8—30 % of the PHCs in the study area had the full complement of staff required to provide maternal and newborn care as per IPHS (Indian Public Health Standards) norms. On assessments of knowledge and skills among auxiliary nurse midwives (ANMs) in PHCs, it was observed that their scores on the three major diseases of importance in the neonatal period in India were <15 % (on LBW/prematurity), <5 % (on birth asphyxia) and <5 % (on neonatal infections). Similarly, there were also deficiencies in the availability of drugs, equipment and infrastructure for newborn care (data not shown). In the private sector, it was seen that there was no major differentiation into BEmNC and CEmNC centres, with facilities offering basic care also offering comprehensive care.

These findings have major implications. While it is known that ‘distal’ determinants of child health such as health and nutrition of adolescent girls, and of pregnant and lactating mothers do require attention, there is also the need, in parallel, for governments to act on ‘proximate health system’ determinants in the short-term. At the population-level, there are differences between and within states in India, with neonatal *versus* post-neonatal causes of mortality operating predominantly in different regions [16, 17]. In states with high levels of infant mortality, both neonatal and postneonatal disorders are common, while in states with relatively lower levels of infant mortality, neonatal causes of death predominate. In addition to intervening at the level of distal determinants, governments also need to pay attention to strengthening of health facilities at district and *taluk* levels to be able to provide quality newborn care through the provision of emergency signal functions for sick newborns.

Several human resource recruitment strategies may need to be adopted to tackle the problem of shortage of doctors. Temporary measures such as ‘contracting-in’ of obstetrics specialists or training of generalist medical officers in ‘emergency obstetric care’ have been attempted to address shortcomings in provision of the signal functions for obstetric care but with limited success [18, 19]. Other innovative strategies such as ‘redistribution’ of doctors may need to be considered so that there are adequate numbers posted in government hospitals (at district and *taluk *levels) on a priority basis instead of spreading them thinly across all ‘small’ and ‘large’ government sector hospitals. This is especially critical given that the Indian government has identified all district and *taluk* hospitals along with select CHCs for provision of comprehensive emergency care, the remaining CHCs and all 24/7 PHCs for provision of basic emergency care, and the peripheral level non-24/7 PHCs and health subcentres for provision of routine essential care [20].

There must also be on-going attempts at developing newer approaches to facility-wide quality improvement and newborn care training strategies given that available evidence points to limited usefulness of current models of ‘in-service’ training programs [21, 22]. Large-scale, region-wide on-site mentoring and supportive supervision of auxiliary nurse midwives (ANMs) may be an optimal approach to improving the quality of health care provided in public sector facilities [23]. Supportive supervision that makes use of staff self-assessments, quality improvement tools and processes, structured case-sheets for documentation and quality audits can help improve provider-preparedness and facility-readiness for maternal and newborn health (MNH) service delivery. It is an effective substitute to the one-off, traditional, hierarchical supervisory visits that ANMs and other staff are usually exposed to from their line-managers in their routine work. Compared to obstetric procedures however, there is caution to be exercised in the extent of ‘task-shifting’ that can safely be executed for neonatal life-saving procedures such as resuscitation, antibiotic administration and initiation/maintenance of kangaroo mother care by ANMs in health facilities. There is insufficient evidence till date on the effectiveness of delivery of these services by ANMs; there is a need for well-functioning referral systems to be in place as a pre-condition as well as to validate clinical treatment algorithms used by ANMs in the delivery of these services [24].

Given that most of the newborn care services are located within the private sector, building public-private partnerships to improve availability may seem like an option. A review of sixteen case studies of such partnerships from across nine states in India has however revealed that successful partnerships have been with private, not-for-profit organizations and that partnership with the private sector is not a substitute for the provision of health services by the public sector [25].

Furthermore, the concept of equity in health needs to be operationalized in primary health care through efforts aimed at reducing disparities in access to key neonatal care services. While governments routinely express explicit targets for increasing institutional deliveries, there are no such explicit targets for assessing and improving quality of neonatal care. We have earlier identified deficits in the availability and distribution of emergency obstetric care in the same geographic region suggesting that equity gaps in maternal and newborn care go hand-in-hand in the northern part of Karnataka state [7].

Our study has a number of limitations. Firstly, for population-level calculations we have considered our study area as a ‘closed jurisdiction’. In practice, hospitals in the study districts cater to patients from outside the study area and similarly patients from the study area can access hospitals located outside the study districts. However, excluding the seasonal mobility of female sex workers from this region [26], there is no known large-scale migration in the general population. Secondly, though we covered almost all the public sector health facilities in the study area, a substantial proportion of private health facilities had to be excluded from the analysis owing to their ‘non-responder’ status. If the latter were not providing EmNC, then the true differences between public and private sector facilities would be narrower. Thirdly, our selection criteria of signal functions for basic and comprehensive care are slightly different from those proposed by others [9]; in addition, we used a combination of self-reporting supplemented with record review and direct observation to assess signal function provision by health facilities. Differences in assessment criteria and study methods could potentially affect study results and impact comparisons between regions [9, 27]. Though functionality of facilities may need to be assessed in a more in-depth manner, our methodology is probably sufficient for studying differences between hospitals and regions and also over time.

## Conclusion

In summary, describing the availability of emergency neonatal care by geographic location and ownership of health facilities is key to understanding access and equity considerations in a region. Key policy implications include an equity case or public health rationale for targeting disadvantaged regions or populations, re-distribution of healthcare professionals for optimization of emergency neonatal care services in the face of shortage, innovative capacity-building and systems strengthening approaches, and targeted spending to remove bottlenecks. Future research should also look at the appropriateness of these signal functions as well as benchmarks for population-level measurement, and relate them to neonatal survival [9]. In the meantime, efforts to immediately improve the provision of emergency neonatal care services in areas of need such as the districts in our study should be pursued.
